# Predicting postoperative rehemorrhage in hypertensive intracerebral hemorrhage using noncontrast CT radiomics and clinical data with an interpretable machine learning approach

**DOI:** 10.1038/s41598-024-60463-2

**Published:** 2024-04-27

**Authors:** Weigong Wang, Jinlong Dai, Jibo Li, Xiangyang Du

**Affiliations:** Department of Neurosurgery, Lu’an Hospital of Traditional Chinese Medicine, No. 76 Renmin Road, Jin’an District, Lu’an, 237000 Anhui China

**Keywords:** Machine learning, Radiomics, Prediction, Postoperative rehemorrhage, Hypertensive intracerebral hemorrhage, Stroke, Stroke

## Abstract

In hypertensive intracerebral hemorrhage (HICH) patients, while emergency surgeries effectively reduce intracranial pressure and hematoma volume, their significant risk of causing postoperative rehemorrhage necessitates early detection and management to improve patient prognosis. This study sought to develop and validate machine learning (ML) models leveraging clinical data and noncontrast CT radiomics to pinpoint patients at risk of postoperative rehemorrhage, equipping clinicians with an early detection tool for prompt intervention. The study conducted a retrospective analysis on 609 HICH patients, dividing them into training and external verification cohorts. These patients were categorized into groups with and without postoperative rehemorrhage. Radiomics features from noncontrast CT images were extracted, standardized, and employed to create several ML models. These models underwent internal validation using both radiomics and clinical data, with the best model’s feature significance assessed via the Shapley additive explanations (SHAP) method, then externally validated. In the study of 609 patients, postoperative rehemorrhage rates were similar in the training (18.8%, 80/426) and external verification (17.5%, 32/183) cohorts. Six significant noncontrast CT radiomics features were identified, with the support vector machine (SVM) model outperforming others in both internal and external validations. SHAP analysis highlighted five critical predictors of postoperative rehemorrhage risk, encompassing three radiomics features from noncontrast CT and two clinical data indicators. This study highlights the effectiveness of an SVM model combining radiomics features from noncontrast CT and clinical parameters in predicting postoperative rehemorrhage among HICH patients. This approach enables timely and effective interventions, thereby improving patient outcomes.

## Introduction

The modern lifestyle has led to a surge in hypertension prevalence, a condition that poses significant risks to the cardiovascular system^1,2^. A common and severe complication of hypertension is hypertensive intracerebral hemorrhage (HICH), characterized by its sudden onset, rapid progression, and associated complications^3,4^. HICH, a subtype of spontaneous intracerebral hemorrhage (sICH), has the highest mortality rate among cerebrovascular diseases^5^. HICH accounts for about 30% of stroke incidences^6^. Currently, there is no specific treatment for HICH. However, emergency surgical interventions, such as craniotomy with hematoma evacuation, have shown potential in reducing mortality rates^7^. This procedure effectively alleviates intracranial pressure and reduces hematoma volume in HICH patients^8^. Despite these benefits, the risk of postoperative rehemorrhage remains a significant concern, often leading to neurological deterioration and increased mortality^9,10^. Thus, the early prediction and identification of postoperative rehemorrhage are critical for improving HICH patient prognosis.

Previous studies highlight the importance of closely examining hematoma characteristics, such as heterogeneity, shape, and volume, in evaluating intracerebral hemorrhage outcomes^11^. Furthermore, recent reports have shown that the spot sign on computed tomography (CT) angiography is an important predictor of rehemorrhage after craniotomy or endoscopic surgery in patients with sICH^12–14^. The spot sign helps to define the vulnerability of sICH, but there are limitations to the use of CT angiography. First, there is no time for the examination because some patients have emergency surgery. Second, an iodinated contrast agent utilized in CT angiography is contraindicated in patients with asthma, kidney dysfunction, or others. Thus, the noncontrast CT marker for rehemorrhage prediction is needed. The noncontrast CT signs, such as blend sign and black hole sign, are strongly related to postoperative rehemorrhage in patients with HICH^15^; however, the recognition of these signs is susceptible to the subjective assessment of doctors^16^.

Radiomics is a noninvasive approach for high-throughput extraction and evaluation of huge amounts of quantitative features from medical images^17^. Its strength is the conversion of visual image information into deep-seated data for quantitative analysis^18^. This method's integration into medical diagnostics has proven invaluable across various fields, including but not limited to oncology and cardiology^19–21^. Recent advancements further highlight its effectiveness in non-invasively predicting outcomes for patients with HICH, showcasing its broad applicability and potential in medical prognostication^5,22,23^. However, the complex and frequently nonlinear connections between the myriad subtle features identified by radiomics and their clinical outcomes pose a substantial analytical challenge. This complexity constrains the efficacy of linear predictive models, like logistic regression (Logit), from reaching optimal predictive precision. In this context, the deployment of machine learning (ML)—a branch of artificial intelligence celebrated for its exceptional ability to decode complex patterns in vast and detailed datasets—is crucial for developing an effective predictive model^24^. Popular ML classifiers, such as random forest (RF) and extreme gradient boosting (XGBoost), have shown their adaptability in applications from detecting intracerebral hemorrhage (ICH) to predicting outcomes in patients with sICH^25,26^. However, there is limited research on ML models that use noncontrast CT radiomics to predict postoperative rehemorrhage in HICH patients.

With this background, our study is dedicated to developing and validating an interpretable ML model that utilizes radiomics features from noncontrast CT scans. Our objective is to forecast the risk of postoperative rehemorrhage in HICH patients following craniotomy, providing clinicians with a tool for early detection and enabling prompt intervention.

## Methods

This study was approved by the Ethics Committee of Lu’an Hospital of Traditional Chinese Medicine (LASZYY-LL-2023013) and carried out in accordance with the Declaration of Helsinki. Due to its retrospective nature, informed consent requirements were waived by the Ethics Committee of Lu’an Hospital of Traditional Chinese Medicine.

### Study population

**Figure 1:**
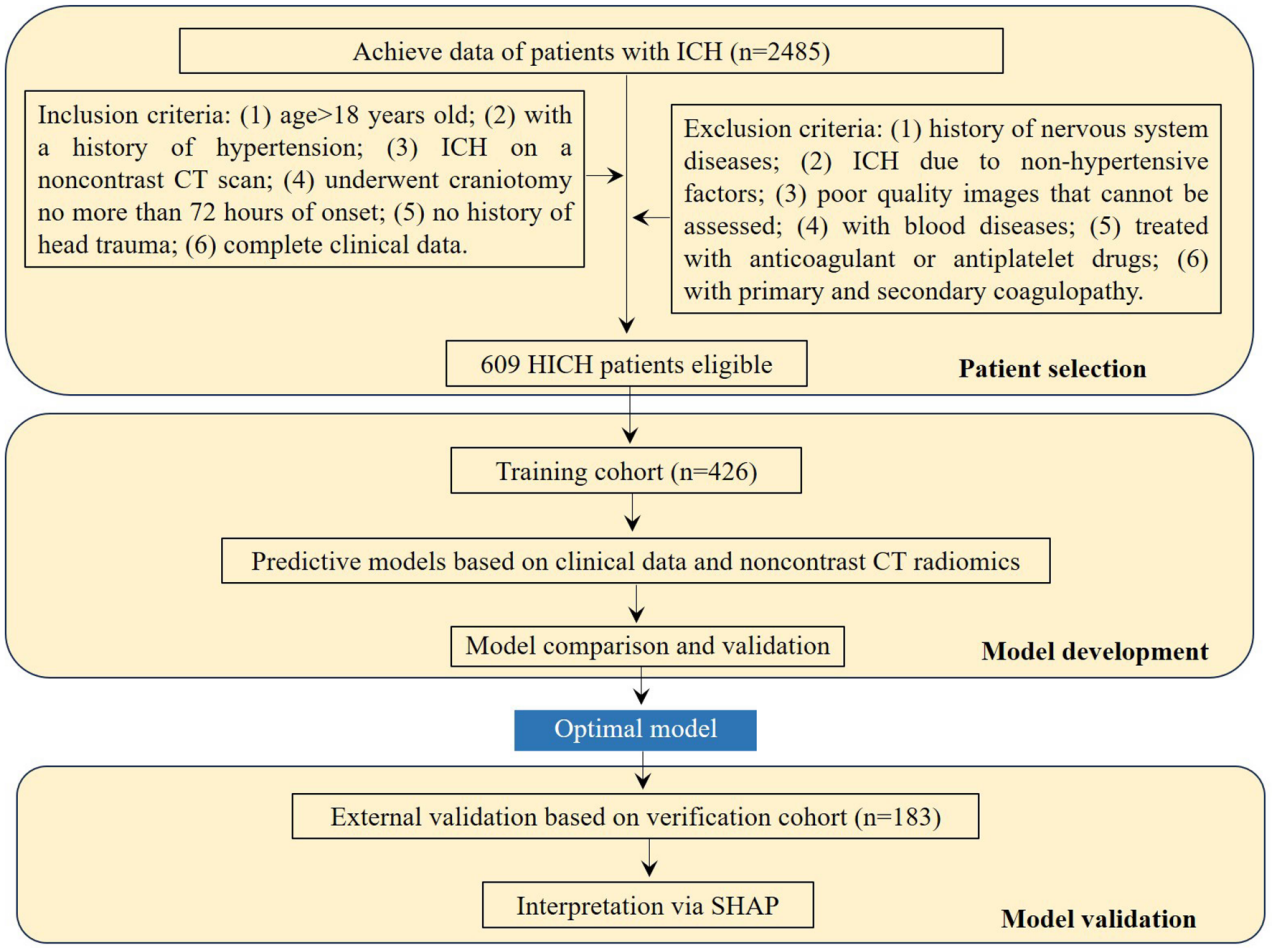
Flowchart for HICH patient selection and cohort distribution for predictive model development and validation. *ICH* intracerebral hemorrhage, *HICH* hypertensive intracerebral hemorrhage, *CT* computed tomography, *SHAP* Shapley additive explanations.

A retrospective review of all patients diagnosed with ICH from Lu’an Hospital of Traditional Chinese Medicine between February 2017 and January 2023 was conducted. ICH was determined using noncontrast CT images showing parenchymal hemorrhage. The inclusion criteria: (1) age > 18 years old; (2) with a history of hypertension; (3) ICH on a noncontrast CT scan; (4) underwent craniotomy no more than 72 h of onset; (5) no history of head trauma; (6) complete clinical data. Exclusion criteria: (1) history of nervous system diseases such as brain tumors, cerebral infarction, cerebral aneurysms and other nervous system diseases; (2) ICH due to non‑hypertensive factors; (3) poor quality images that cannot be assessed; (4) with hemophilia, leukemia, and other blood diseases; (5) treated with anticoagulant or antiplatelet drugs; (6) with primary and secondary coagulopathy. According to the inclusion and exclusion criteria, 609 patients were enrolled in our study. Four hundred and twenty-six patients from February 2017 to August 2021 were involved in the training cohort and 183 patients from September 2021 to January 2023 were assigned to the external verification cohort (Fig. 1). HICH is the rupture and bleeding of vessels of the cerebral parenchyma triggered by continuously elevated or violently fluctuating blood pressure.

For each patient, clinical characteristics and noncontrast CT imaging findings were meticulously documented. The diagnosis of ICH was confirmed by a baseline noncontrast CT scan within 12 h of symptom onset. Craniotomy was conducted within 72 h of onset. After the operation, at least two follow-up CT scans were conducted over the subsequent 3 days. According to the criteria from previously published studies^15,27^, we characterized postoperative rehemorrhage as either an increase in hematoma volume by more than 33% compared to the previous postoperative CT scan (wherein the ICH volume had significantly reduced after surgery) or the reappearance of hyperdensity within the focal region on a follow-up CT scan after its complete surgical removal.

### Imaging acquisition

The baseline noncontrast CT scans (General Electric Medical Systems, Milwaukee, WI, USA) were conducted following standard clinical parameters, utilizing axial sections that were 5 mm in thickness. The images were captured and archived for subsequent analysis. Two experienced neuroimaging experts independently reviewed all the images.

### Image segmentation and feature extraction

**Figure 2:**
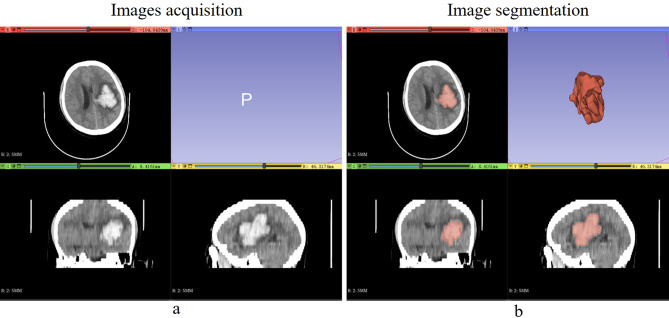
Noncontrast CT images was semi-automatic segmentation. *CT* computed tomography.

Baseline noncontrast CT images, retrieved from the picture archiving and communication system (PACS), were saved as DICOM files. Employing the semi-automatic segmentation software 3D Slicer (Version 5.0.2), an experienced radiologist (R1), without access to clinical data, delineated the regions of interest (ROIs) (Fig. 2). A second radiologist (R2), with over 10 years of experience, independently confirmed these outlines, also without seeing the clinical data, employing the same methodology as R1. The consistency of ROI delineation between observers was assessed through intraclass correlation coefficients (ICCs), with values ≥ 0.80 indicating high reproducibility. Radiomic signatures from each ROI were extracted using PyRadiomics (Version 3.7). Overall, 6 image types and 6 feature classes were obtained.

### Data preprocessing

Before developing the predictive model, a crucial data preprocessing phase was conducted to ensure the unbiasedness of the process. This phase standardized all data, including both radiomics features and clinical information. Standardization varied with the data type: continuous variables were normalized using Z-score to achieve a mean of zero and a standard deviation of one, whereas categorical variables were converted into binary form, labeled as “0” or “1”.

### Selection of radiomics features

We executed 3 feature selection steps to mitigate overfitting. Initially, ICCs exceeding 0.8 were deemed indicative of satisfactory agreement. Subsequently, a *t*-test was applied to each feature to differentiate between patients with postoperative rehemorrhage and those without. Lastly, employing the least absolute shrinkage and selection operator (LASSO) logistic regression coupled with tenfold cross-validation, features related to postoperative rehemorrhage with non-zero coefficients were selected from those exhibiting P-values less than 0.05 in the *t*-test.

### Derivation and internal validation of ML models

To assess the risk of postoperative rehemorrhage, we utilized four established ML classifiers: Logit, RF, support vector machine (SVM), and XGBoost. We crafted distinct prediction models using clinical data, radiomics features, and a combination thereof. During training, we applied a triply-repeated five-fold cross-validation to enhance data utilization, segmenting the training set into inner training and test subsets for sequential evaluation. For the RF model, we selected a configuration of 500 trees and determined the number of features for node splitting based on the square root of the total number of features. The SVM used a radial basis function (RBF) kernel, adept at handling non-linear data, with hyperparameters finely adjusted via grid search, including cost parameters across [0.1, 1, 10] and gamma parameters for the RBF kernel within [0.001, 0.01, 0.1]. XGBoost parameters, such as a 0.02 learning rate, a maximum tree depth of 4, and a 600-tree ensemble, were optimized through grid search to ensure a delicate balance between complexity and accuracy in predictions, streamlining the model development process.

After deriving each model, we subjected them to a stringent internal validation process to assess their discrimination, calibration, and clinical applicability. The selection of the optimal predictive model was based on its outstanding discriminatory power, alongside robust calibration and clinical utility.

### Interpretability and external validation of ML models

After identifying the optimal predictive models, our focus shifted to understanding the contribution of each variable to the prediction. We incorporated the SHAP (Shapley Additive Explanation) methodology to gain a deeper insight into feature importance, emphasizing the most influential variables. Features were ranked by their SHAP values in descending order of influence, pinpointing the key predictors within our patient cohort. To ensure the models' robustness, we conducted external validation. This thorough assessment affirmed their discriminative power, calibration, and clinical relevance, offering a well-rounded perspective on the predictive strength of these models.

### Statistical analysis

Statistical evaluations were conducted using R statistical software (Version 4.2.1) and Python programming software (Version 3.7.1). Continuous variables that exhibited a skewed distribution were presented as median [interquartile range (IQR)] and evaluated with the Mann–Whitney *U*-test. Categorical data were denoted as number (percentage) and analyzed using the χ^2^ test. Model performance evaluation included receiver operating characteristic (ROC) curve analysis, focusing on the area under the curve (AUC), and metrics such as Precision, Recall, and F1 Score to thoroughly evaluate discrimination ability. AUC comparisons employed Delong's test. Model fit was assessed using calibration curve analysis and the Brier Score for probability prediction accuracy. To gauge the clinical utility of the models, decision curve analysis (DCA) was used to calculate net benefits across various threshold probabilities.

### Ethical approval

This study was approved by the Ethics Committee of Lu’an Hospital of Traditional Chinese Medicine (No. LASZYY-LL-2023013).

### Informed consent

Written informed consent was waived by the Ethics Committee of Lu’an Hospital of Traditional Chinese Medicine.

## Results

### Patient summary

Data of 2485 ICH patients were obtained from the inpatients management system. After strict screening based on the inclusion and exclusion criteria, our study ultimately included 609 patients, divided into two cohorts: 426 in the training cohort and 183 in the externally verification cohort, as detailed in Fig. 1. The prevalence of postoperative rehemorrhage was comparable between the training (18.8%, 80/426) and externally verification (17.5%, 32/183) cohorts, with no significant statistical difference observed (χ^2^ = 0.143, P = 0.706). Supplementary material 1 further supports these findings, confirming uniform distribution across both cohorts without significant variations in clinical characteristics (all P > 0.05).

### Comparative clinical characteristics of patients with and without postoperative rehemorrhage in the training cohort

Table 1 compares clinical characteristics between patients with and without postoperative rehemorrhage in the training cohort. It shows that higher rehemorrhage risk is associated with increased baseline HICH volume, SBP at admission, time to surgery, and irregular hematoma shape (all P < 0.05). Key clinical parameters were standardized using Z-score normalization to a mean of zero and a standard deviation of one. These standardized metrics were recorded for the development of clinical ML prediction models.

**Table 1:** Comparative clinical characteristics of patients with and without rehemorrhage.

Variable	Non-rehemorrhage group (N = 346)	Rehemorrhage group (N = 80)	P value
Age, years, median (IQR)	59.00 (50.00, 70.00)	61.00 (51.00, 69.00)	0.868*
Gender, n (%)			0.840^#^
Female	141 (40.8)	31 (38.7)	
Male	205 (59.2)	49 (61.3)	
Baseline HICH volume, mL, median (IQR)	58.00 (43.25, 68.00)	62.00 (41.75, 79.00)	0.041*
History of smoking, n (%)			0.273^#^
No	249 (72.0)	52 (65.0)	
Yes	97 (28.0)	28 (35.0)	
History of drinking, n (%)			0.913^#^
No	178 (51.4)	40 (50.0)	
Yes	168 (48.6)	40 (50.0)	
History of diabetes mellitus, n (%)			0.469^#^
No	299 (86.4)	66 (82.5)	
Yes	47 (13.6)	14 (17.5)	
DBP on admission, mmHg, n (%)			0.283^#^
< 120	190 (54.9)	38 (47.5)	
≥ 120	156 (45.1)	42 (52.5)	
SBP on admission, mmHg, n (%)			0.022^#^
< 200	155 (44.8)	24 (30.0)	
≥ 200	191 (55.2)	56 (70.0)	
GCS on admission, points, n (%)			0.875^#^
≤ 8	154 (44.5)	37 (46.2)	
> 8	192 (55.5)	43 (53.8)	
Hemorrhage localization, n (%)			0.663^#^
Basal ganglia	154 (44.5)	30 (37.5)	
Ventricle	34 (9.8)	10 (12.5)	
Cerebral lobe	16 (4.6)	5 (6.2)	
Thalamus	78 (22.5)	22 (27.5)	
Cerebellum	64 (18.5)	13 (16.2)	
Shape of hematoma, n (%)			< 0.001^#^
Regular	215 (62.1)	23 (28.8)	
Irregular	131 (37.9)	57 (71.2)	
Time to surgery, hours, n (%)			< 0.001^#^
≤ 6	140 (40.5)	64 (80.0)	
> 6	206 (59.5)	16 (20.0)	
Duration of surgery, hours, median (IQR)	2.60 (1.90, 3.38)	2.75 (2.00, 3.42)	0.383*
Intraoperative blood loss, mL, median (IQR)	203.00 (150.25, 265.75)	214.00 (149.75, 261.25)	0.519*
Rate of hematoma evacuation, %, median (IQR)	84.80 (77.62, 91.45)	85.20 (78.02, 92.80)	0.446*
Platelets, 10^9^/L, median (IQR)	181.00 (137.00, 221.00)	186.00 (141.75, 224.00)	0.659*
APTT, s, median (IQR)	30.80 (26.52, 33.88)	31.10 (27.87, 33.55)	0.535*
INR, median (IQR)	1.11 (0.96, 1.30)	1.13 (0.97, 1.30)	0.568*
Fibrinogen, g/L, median (IQR)	2.94 (2.51, 3.45)	2.92 (2.45, 3.60)	0.577*

^#^For Chi-square test; *For Mann–Whitney *U* test. *IQR* inter-quartile range, *HICH* hypertensive intracerebral hemorrhage, *DBP* diastolic blood pressure, *SBP* systolic blood pressure, *GCS* Glasgow coma scale, *APTT* activated partial thromboplastin time, *INR* international normalized ratio.

### Radiomics analysis

**Figure 3:**
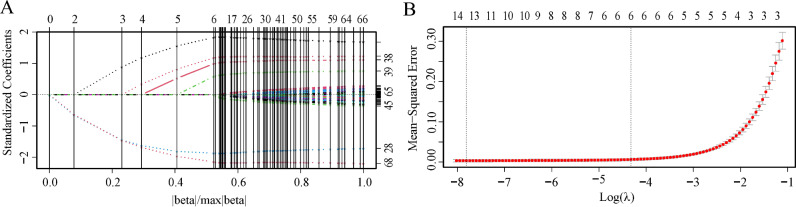
Radiomics feature selection using LASSO logistic regression. (**A**) LASSO coefficient distribution of the 66 radiomics features. (**B**) Selection of the tuning parameter (λ) using tenfold cross validation via the minimum criteria (λ.min) and the 1-standard error of the minimum criteria (λ.1-SE). The optimal λ results in 6 features with nonzero coefficients. *LASSO* least absolute shrinkage and selection operator, *λ* penalty regularization parameter, *SE* standard error.

In the training cohort, we extracted and normalized 1316 radiomics features from each baseline noncontrast CT image. These features exhibited ICCs values spanning from 0.5 to 0.99. Notably, 1152 features (accounting for 87.5%) with an intra-observer ICC of ≥ 0.8 were initially chosen. These were then narrowed down to 66 potential predictors through a Student’s *t*-test. From these, a LASSO logistic regression model pinpointed just 6 optimal features associated with postoperative rehemorrhage, each characterized by non-zero coefficients (Fig. 3A,B).

### Model comparison for postoperative rehemorrhage risk prediction

**Figure 4:**
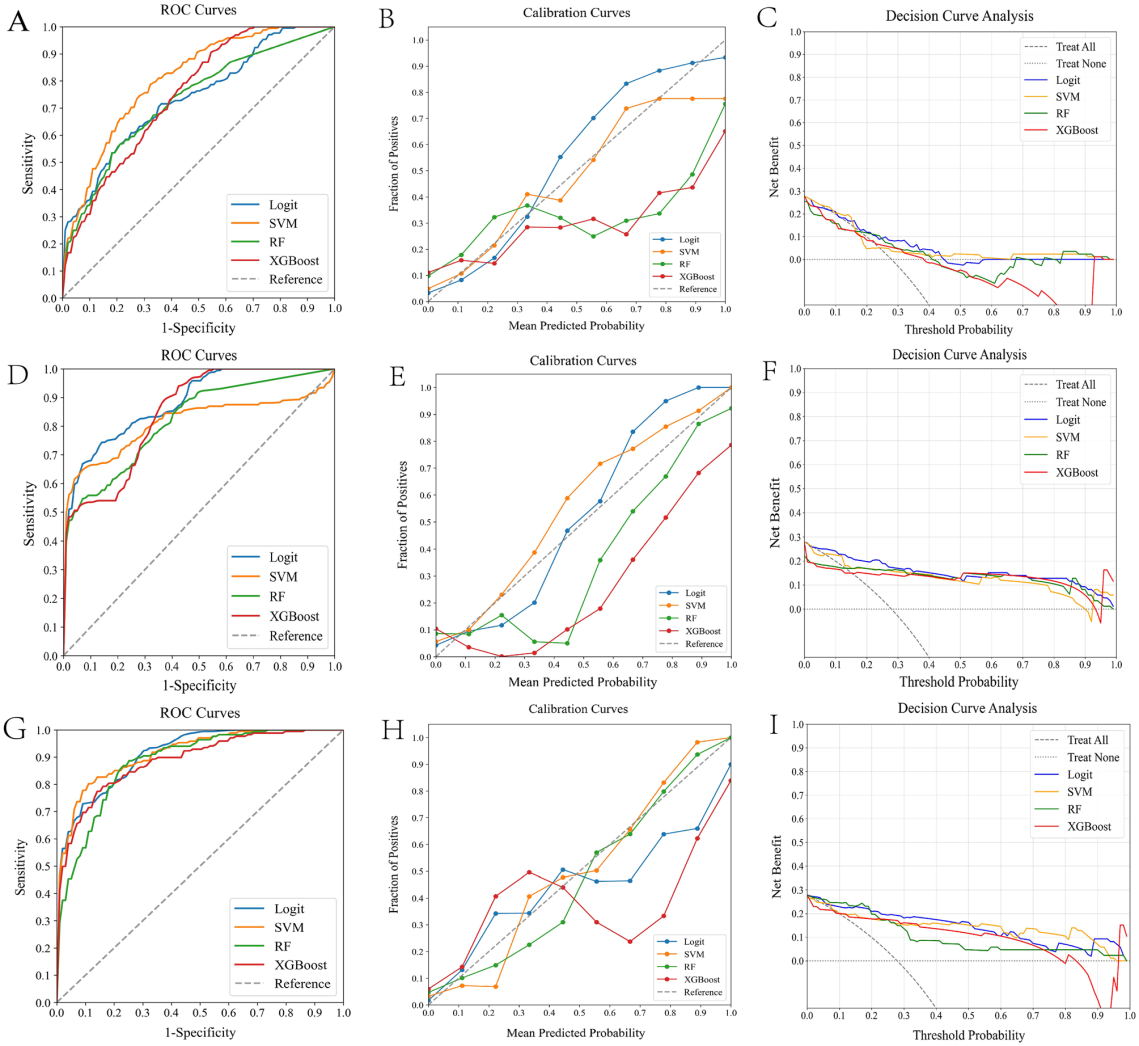
Comparative analysis of ML classifiers-namely Logit, SVM, RF, and XGBoost-across different data types. Figures (**A**–**C**) reveal the performance of these ML classifiers on clinical data, as shown through ROC curves, calibration plots, and DCA. They achieved ROC-AUCs of 0.735, 0.806, 0.733, and 0.749, respectively. Figures (**D**–**F**) focus on the performance of these classifiers with radiomics features, where they yielded AUCs of 0.883, 0.812, 0.821, and 0.845. Figures (**G**–**I**) highlight their performance using combined clinical and radiomics data, achieving AUCs of 0.889, 0.914, 0.883, and 0.887. *ML* machine learning, *ROC* receiver operating characteristic, *AUC* area under the curve, *DCA* decision curve analysis, *Logit* logistic regression, *SVM* support vector machine, *RF* random forest, *XGBoost* extreme gradient boosting.

In our study, we evaluated the effectiveness of predictive models for assessing postoperative rehemorrhage risk in HICH patients post-craniotomy, utilizing four ML classifiers: Logit, SVM, RF, and XGBoost. These classifiers were tested on three distinct datasets: clinical, radiomics, and a combined dataset. Table 2 presents a systematic comparison of these models, with their performance metrics including ROC, calibration, and DCA curves illustrated in Fig. 4. Our findings indicated that models integrating clinical and radiomics data (clinical-radiomics models) significantly outperformed those based solely on clinical (AUC: 0.733–0.806) or radiomics data (AUC: 0.812–0.883), achieving AUCs ranging from 0.883 to 0.914, as confirmed by Delong's test (all P < 0.05).

**Table 2:** Performance of ML classifiers for predicting postoperative rehemorrhage risk using clinical data, radiomics features, and combined datasets in HICH patients.

Data type	ML classifier	AUC	Precision	Recall	F1 score	Brier score
Clinical data	Logit	0.735	0.333	0.083	0.133	0.008
	SVM	0.806	0.288	0.369	0.332	0.006
	RF	0.733	0.368	0.292	0.326	0.061
	XGBoost	0.749	0.389	0.292	0.333	0.073
Radiomics feature	Logit	0.883	0.789	0.625	0.698	0.010
	SVM	0.812	0.923	0.500	0.649	0.007
	RF	0.821	0.789	0.625	0.698	0.032
	XGBoost	0.845	0.824	0.583	0.683	0.068
Combined clinical and radiomics data	Logit	0.889	0.910	0.718	0.756	0.015
	SVM	0.914	0.945	0.711	0.770	0.001
	RF	0.883	0.810	0.712	0.756	0.005
	XGBoost	0.887	0.850	0.708	0.773	0.024

*ML* machine learning, *HICH* hypertensive intracerebral hemorrhage, *AUC* area under the curve, *Logit* logistic regression, *SVM* support vector machine, *RF* random forest, *XGBoost* extreme gradient boosting.

In evaluating clinical-radiomics models, SVM not only achieved the highest AUC score of 0.914 but also exhibited superior calibration, especially noticeable around the 70% threshold. Across all models, performance was consistent in DCA. SVM’s uniform excellence in key metrics, including Precision, Recall, F1 Score, and Brier Score, underscores its effectiveness. This evidence positions SVM as the optimal model for predicting postoperative rehemorrhage risk.

### Assessing ML model with the external verification cohort

**Figure 5:**
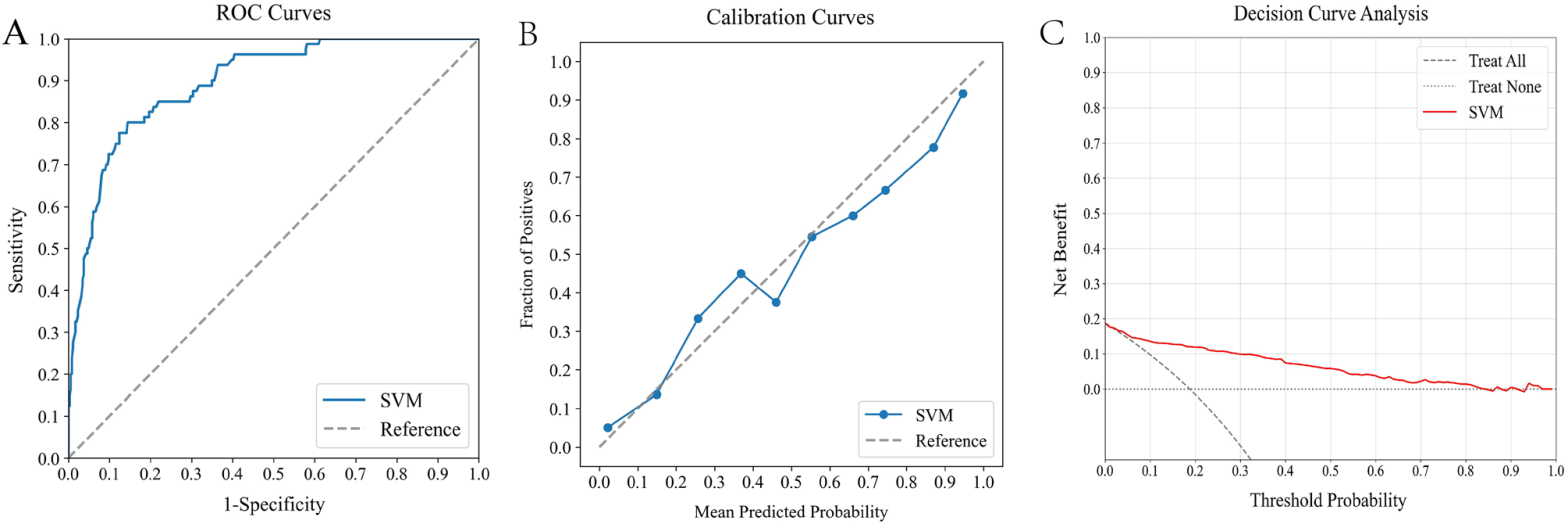
Evaluating the predictive performance of an optimal ML model using an external verification cohort. Figure (**A**) exhibits the ROC curve, which has an AUC of 0.895. Figure (**B**) reveals the calibration curve, showing a strong concordance between the model's predicted probabilities and the actual observed events. Figure (**C**) depicts the DCA, highlighting the net clinical benefit provided by the model. *ML* machine learning, *ROC* receiver operating characteristic, *AUC* area under the curve, *DCA* decision curve analysis.

The external verification cohort was used to evaluate the SVM model's predictive accuracy against actual postoperative rehemorrhage outcomes, employing ROC, calibration, and DCA analyses (Fig. 5). Although the SVM model showed a slight decline in performance compared to the training cohort, it still exhibited significant discriminative ability, achieving an AUC of 0.895 (Fig. 5A). The calibration curve displayed strong agreement between the model's predicted risks and the observed frequencies, especially for predictions above 60% (Fig. 5B). The DCA curve further affirmed the model's effectiveness, highlighting its substantial net benefits (Fig. 5C). These findings underscored the SVM model's potential as a valuable predictive tool for postoperative rehemorrhage risk, underscoring its applicability in clinical settings.

### Interpretation of the model

**Figure 6:**
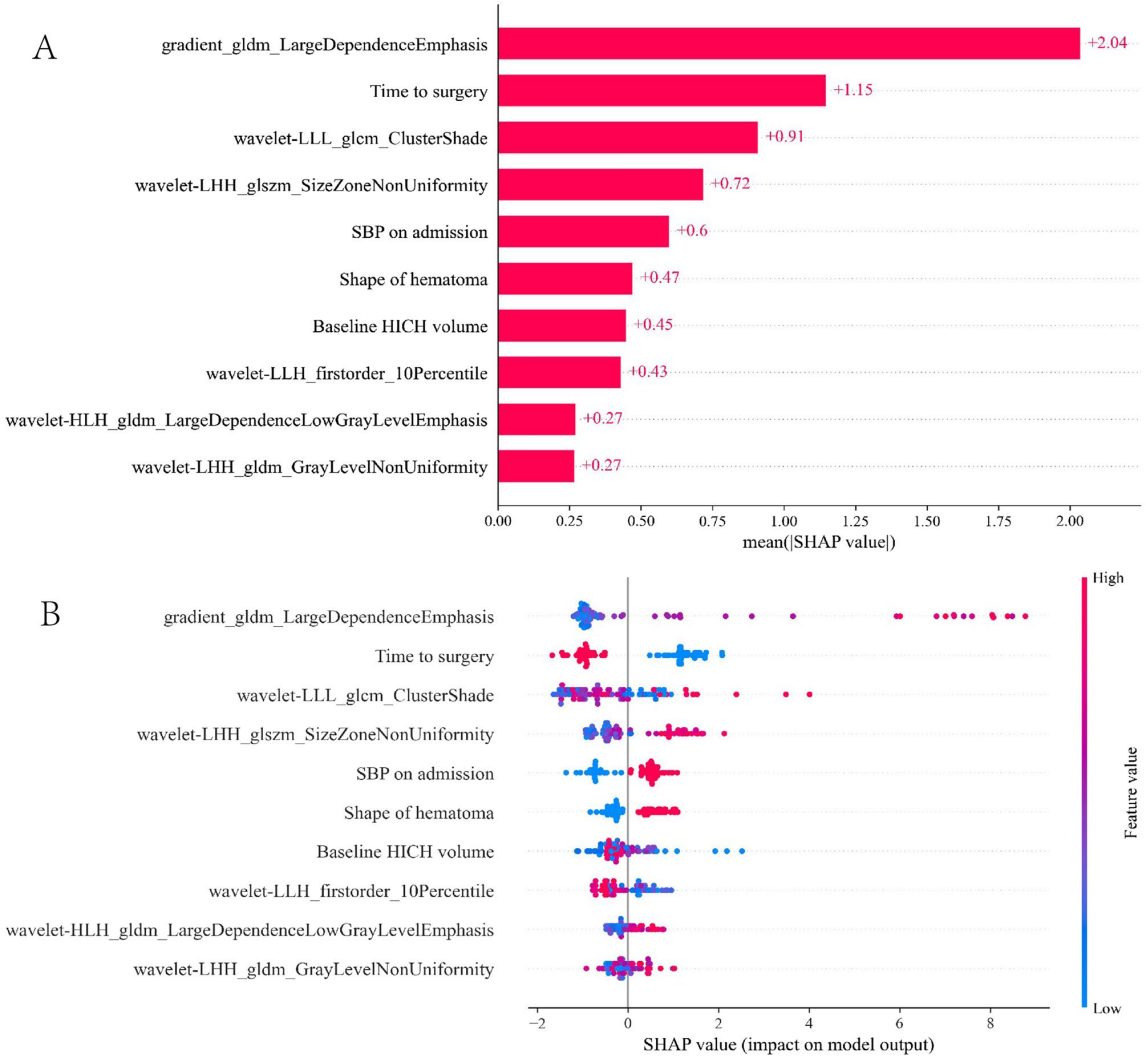
SHAP analysis of the SVM model for predicting postoperative rehemorrhage in HICH patients. Figure (**A**) illustrates the ranking of feature significance as determined by absolute mean SHAP values. Figure (**B**) presents a summary plot incorporating SHAP values, providing a comprehensive visualization of the cumulative influence of each feature. *SHAP* Shapley additive explanation, *SVM* support vector machine, *HICH* hypertensive intracerebral hemorrhage.

The SHAP analysis was utilized to decipher the SVM model, quantifying the impact of each feature. By computing the absolute mean SHAP values, it facilitated the prioritization of features based on their importance. Notably, three radiomics features from baseline noncontrast CT scans and two clinical variables emerged as the most significant influencers in the model (Fig. 6A). A summary plot illustrated the collective impact of these features, represented through their SHAP values (Fig. 6B). This visualization provided comprehensive insights into how each feature contributes to the prediction for individual patients. Importantly, higher values of these top five features correlated with a greater risk of postoperative rehemorrhage in HICH patients following craniotomy.

## Discussion

In our study, we focused on enhancing predictive models for postoperative rehemorrhage in HICH patients post-craniotomy, employing four ML classifiers and analyzing both clinical and noncontrast CT radiomics data. Our thorough assessment, encompassing evaluations of discriminative capacity, calibration, and clinical applicability, established the SVM model, which combines noncontrast CT radiomics with clinical data, as the superior choice. Incorporating SHAP analysis improved the SVM model's interpretability, emphasizing crucial clinical and radiomics predictors of rehemorrhage risk. This innovative method combines noncontrast CT radiomics and clinical data through ML to predict rehemorrhage risk accurately, promoting early and personalized clinical interventions that could notably improve patient outcomes.

HICH, recognized as the most lethal cerebrovascular condition and a subtype of sICH^5^, poses significant treatment challenges. Emergency interventions like craniotomy are effective in reducing mortality by decreasing intracranial pressure and the size of the hematoma^7,8^. However, these patients face a substantial risk of postoperative rehemorrhage, leading to adverse outcomes such as increased in-hospital mortality^28^. Research indicates that post-craniotomy, patients enter an antithrombotic state due to thrombocytopenia, coagulopathy, and the use of antiplatelet and anticoagulant medications, escalating the risk of further intracranial hemorrhagic events^29^. This underscores the critical importance of prompt detection and management to improve patient prognosis.

In our study, we preferred ML models for their ability to navigate complex non-linear relationships between variables and outcomes, outperforming traditional linear predictive approaches^30^. We tested four ML models on both clinical and radiomics data, finding that all achieved satisfactory calibration and clinical utility, though they varied significantly in their ability to discriminate. Notably, the combined clinical-radiomics ML models proved most effective in predicting the risk of postoperative rehemorrhage, offering superior discrimination capabilities. This superiority likely originates from the extensive combination of clinical and radiomics features, offering a wider analytical foundation than models based solely on clinical or radiomics data. These differences in feature integration could account for the variations in predictive accuracy observed.

Within our selection of ML models, SVM emerged as the most efficacious clinical-radiomics model, demonstrating high accuracy even during external validation. To tackle the interpretability challenges inherent in complex ML models, we utilized SHAP methodology. This technique elucidates the decision-making process at the cohort level, enhanced by intuitive visualizations, allowing for a nuanced understanding of how individual variables influence predictions, thus building trust in AI among clinicians^31,32^. It identified five principal predictors of postoperative rehemorrhage risk: three radiomics features from noncontrast CT scans and two clinical factors. The significance of noncontrast CT radiomics features was anticipated due to their correlation with rehemorrhage risk. These features, detailed by radiomics, provide a more comprehensive and objective assessment than traditional imaging alone. While the biological correlation of certain texture features may seem abstract at first glance, these characteristics are instrumental in delineating the complex nature of hematoma beyond basic parameters like shape and volume. Additionally, admission SBP and time to surgery were confirmed as crucial clinical predictors, consistent with evidence that blood pressure variability impacts rehemorrhage risk^33^. Rigorous blood pressure monitoring, with real-time and dynamic assessments, is essential for effective management. The first 6 h after onset are pivotal, with unstable hemostasis due to hematoma pressure on ruptured vessels heightening the risk of rehemorrhage post-surgery. Postponing surgery after 6 h markedly reduces these risks, underscoring the critical nature of surgical timing^34^. Together with SHAP, SVM offers a detailed insight into the impact of variables on outcomes, proving invaluable for predicting postoperative rehemorrhage and enhancing the role of ML in clinical decision-making and improving patient outcomes.

Our study presented 3 limitations. Firstly, our investigation was conducted using a retrospective design. To ensure the generalizability and validity of the ML model, prospective studies are warranted. Secondly, the clinical relevance of these AI-generated features might be challenging to interpret; however, advancements in radiomics and visualization tools are bridging this gap, enhancing our understanding and integration of these technologies into clinical practice^35,36^. Efforts to address the mentioned shortcomings are continuously underway^37–39^. Thirdly, the limited sample size constituted another limitation, underscoring the need for further studies with larger cohorts to corroborate the predictive potential of our findings.

## Conclusions

In conclusion, our evaluation identified the SVM model, integrating noncontrast CT radiomics and clinical data, as the most effective ML approach for predicting postoperative rehemorrhage in HICH patients. This innovative combination of noncontrast CT radiomics with clinical data through ML, particularly our refined SVM model, promises to enhance the accuracy of postoperative rehemorrhage risk assessment. Anticipated to support clinicians in decision-making for high-risk patients, this development could lead to early, personalized interventions, significantly improving patient outcomes.

### Supplementary Information

Supplementary Information 1.

Supplementary Information 2.

## Data Availability

All data generated or analyzed during this study are included in this article. Further enquiries can be directed to the corresponding author.
