# Supplementary material for: Predicting postoperative rehemorrhage in hypertensive intracerebral hemorrhage using noncontrast CT radiomics and clinical data with an interpretable machine learning approach
Source: Sci Rep. 2024 Apr 27;14:9717. doi: 10.1038/s41598-024-60463-2 (PMC11055901; doi:10.1038/s41598-024-60463-2)
Supplement: Supplementary file 1 — Supplementary Information 1. [file 41598_2024_60463_MOESM1_ESM.docx]

**Supplemental Table 1** Comparisons of the clinical characteristics between the training and externally verification cohorts

| Variable | Training cohort  (N=426) | Externally verification cohort  (N=183) | P value |
| --- | --- | --- | --- |
| Age, years, median (IQR) | 59.00 (50.00, 70.00) | 58.00 (48.00, 68.00) | 0.205^*^ |
| Gender, n(%) |  |  | 0.983^#^ |
| Female | 172 (40.4) | 73 (39.9) |  |
| Male | 254 (59.6) | 110 (60.1) |  |
| Baseline HICH volume, ml, median (IQR) | 58.50 (43.00, 70.00) | 56.00 (42.00, 71.00) | 0.588^*^ |
| History of smoking, n (%) |  |  | 0.586^#^ |
| No | 301 (70.7) | 134 (73.2) |  |
| Yes | 125 (29.3) | 49 (26.8) |  |
| History of drinking, n (%) |  |  | 0.908^#^ |
| No | 218 (51.2) | 92 (50.3) |  |
| Yes | 208 (48.8) | 91 (49.7) |  |
| History of diabetes mellitus, n (%) |  |  | 0.989^#^ |
| No | 365 (85.7) | 156 (85.2) |  |
| Yes | 61 (14.3) | 27 (14.8) |  |
| DBP on admission, mmHg, n (%) |  |  | 0.977^#^ |
| <120 | 228 (53.5) | 97 (53.0) |  |
| ≥120 | 198 (46.5) | 86 (47.0) |  |
| SBP on admission, mmHg, n (%) |  |  | 0.765^#^ |
| <200 | 179 (42.0) | 80 (43.7) |  |
| ≥200 | 247 (58.0) | 103 (56.3) |  |
| GCS on admission, points, n (%) |  |  | 0.967^#^ |
| ≤8 | 191 (44.8) | 81 (44.3) |  |
| >8 | 235 (55.2) | 102 (55.7) |  |
| Hemorrhage localization, n (%) |  |  | 0.886^#^ |
| Basal ganglia | 184 (43.2) | 82 (44.8) |  |
| Ventricle | 44 (10.3) | 21 (11.5) |  |
| Cerebral lobe | 21 (4.9) | 10 (5.5) |  |
| Thalamus | 100 (23.5) | 36 (19.7) |  |
| Cerebellum | 77 (18.1) | 34 (18.6) |  |
| Shape of hematoma, n (%) |  |  | 0.315^#^ |
| Regular | 238 (55.9) | 111 (60.7) |  |
| Irregular | 188 (44.1) | 72 (39.3) |  |
| Time to surgery, hours, n (%) |  |  | 0.812^#^ |
| ≤6 | 204 (47.9) | 85 (46.4) |  |
| >6 | 222 (52.1) | 98 (53.6) |  |
| Duration of surgery, hours, median (IQR) | 2.60 (1.90, 3.40) | 2.50 (1.80, 3.20) | 0.241^*^ |
| Intraoperative blood loss, ml, median (IQR) | 206.00 (150.25, 265.00) | 217.00 (158.00, 270.50) | 0.219^*^ |
| Rate of hematoma evacuation, %, median (IQR) | 84.80 (77.80, 92.00) | 83.8 (76.70, 91.00) | 0.213^*^ |
| Platelets, 10^9^/L, median (IQR) | 181.50 (138.00, 221.00) | 184.00 (141.00, 220.00) | 0.711^*^ |
| APTT, s, median (IQR) | 30.90 (26.90, 33.80) | 29.80 (26.05, 33.80) | 0.174^*^ |
| INR, median (IQR) | 1.11 (0.97, 1.30) | 1.16 (1.00, 1.30) | 0.168^*^ |
| Fibrinogen, g/L, median (IQR) | 2.94 (2.50, 3.46) | 3.04 (2.44, 3.52) | 0.431^*^ |

^#^, for chi-square test; ^*^, for Mann-Whitney U-test. IQR, inter-quartile range; HICH, hypertensive intracerebral hemorrhage; DBP, diastolic blood pressure; SBP, systolic blood pressure; GCS, glasgow coma scale; APTT, activated partial thromboplastin time; INR, international normalized ratio.
