# Supplementary material for: Predicting postoperative rehemorrhage in hypertensive intracerebral hemorrhage using noncontrast CT radiomics and clinical data with an interpretable machine learning approach
Source: Sci Rep. 2024 Apr 27;14:9717. doi: 10.1038/s41598-024-60463-2 (PMC11055901; doi:10.1038/s41598-024-60463-2)
Supplement: Supplementary file 2 — Supplementary Information 2. [file 41598_2024_60463_MOESM2_ESM.pdf]

```
root = tk.Tk()
root.withdraw()
file_path = filedialog.askopenfilename(filetypes=[("Excel files", "*.xlsx")])
data = pd.read_excel(file_path)


import pandas as pd
import numpy as np
import matplotlib.pyplot as plt
from sklearn.model_selection import train_test_split, StratifiedKFold, RepeatedStratifiedKFold
from sklearn.linear_model import LogisticRegression
from sklearn.neighbors import KNeighborsClassifier
from xgboost import XGBClassifier
from sklearn.svm import SVC
from sklearn.ensemble import RandomForestClassifier
from sklearn.metrics import roc_curve, auc, accuracy_score, recall_score, confusion_matrix
from sklearn.calibration import calibration_curve, CalibratedClassifierCV
import tkinter as tk
from tkinter import filedialog
import warnings
import matplotlib as mpl
warnings.filterwarnings('ignore')
mpl.rcParams['figure.dpi'] = 300
from sklearn.metrics import precision_score, recall_score, f1_score


X = data.drop(columns=['Group'])
y = data['Group']
X_train, X_test, y_train, y_test = train_test_split(X, y, test_size=0.2, random_state=42)


log_reg = LogisticRegression(max_iter=1000)
svm = SVC(probability=True)
random_forest = RandomForestClassifier()
xgb = XGBClassifier(use_label_encoder=False, eval_metric='logloss')


cv = RepeatedStratifiedKFold(n_splits=5, n_repeats=3, random_state=42)
models = {'Logit': log_reg, 'SVM': svm, 'RF': random_forest, 'XGBoost': xgb}


# Train and evaluate models
plt.figure(figsize=(6, 6))
for model_name, model in models.items():
    print(f"Evaluating {model_name}:")
    # Compute ROC curve and AUC
    tprs = []
    aucs = []
```

```
    mean_fpr = np.linspace(0, 1, 100)
    for train, val in cv.split(X_train, y_train):
        model.fit(X_train.iloc[train], y_train.iloc[train])
        y_pred_prob = model.predict_proba(X_train.iloc[val])[:, 1]
        #if model_name == 'SVM':
            # y_pred_prob = 1 - y_pred_prob
        fpr, tpr, thresholds = roc_curve(y_train.iloc[val], y_pred_prob)
        tprs.append(np.interp(mean_fpr, fpr, tpr))
        tprs[-1][0] = 0.0
        roc_auc = auc(fpr, tpr)
        aucs.append(roc_auc)
    # Plot ROC curve
    mean_tpr = np.mean(tprs, axis=0)
    mean_tpr[-1] = 1.0
    mean_auc = auc(mean_fpr, mean_tpr)
    std_auc = np.std(aucs)
    plt.plot(mean_fpr, mean_tpr, label=f'{model_name} (AUC = {mean_auc:.3f})', 
linewidth=2)
# Set plot properties
plt.plot([0, 1], [0, 1], linestyle='--', lw=2, color='gray', label='Reference', alpha=.8)
plt.xlim([0.0, 1.0])
plt.ylim([0.0, 1.0])
plt.gca().set_aspect('equal', adjustable='box')
plt.xlabel('1-Specificity', fontsize=16, labelpad=10)
plt.ylabel('Sensitivity', fontsize=16, labelpad=10)
plt.title('ROC Curves', fontsize=18, pad=15)
plt.legend(loc='lower right', fontsize=16)
plt.tight_layout()
plt.xticks(np.arange(0, 1.1, 0.1), fontsize=14)
plt.yticks(np.arange(0, 1.1, 0.1), fontsize=14)
# Display and save the plot
plt.savefig('roc_curves.jpg', dpi=300)
plt.show()
metrics = {'Accuracy': accuracy_score, 'Sensitivity': recall_score, 'Precision': 
precision_score, 'F1 Score': f1_score}


for model_name, model in models.items():
    model.fit(X_train, y_train)
    y_pred = model.predict(X_test)
    precision = precision_score(y_test, y_pred)
    recall = recall_score(y_test, y_pred)
    f1 = f1_score(y_test, y_pred)
    print(f"\n{model_name} Model:")
    print(f"Precision: {precision:.3f}")
    print(f"Recall: {recall:.3f}")
    print(f"F1 Score: {f1:.3f}")


plt.figure(figsize=(6, 6))
```

```
for model_name, model in models.items():
    print(f"Evaluating {model_name}:")
    # Compute ROC curve and AUC
    tprs = []
    aucs = []
    mean_fpr = np.linspace(0, 1, 100)
    for train, val in cv.split(X_train, y_train):
        model.fit(X_train.iloc[train], y_train.iloc[train])
        y_pred_prob = model.predict_proba(X_train.iloc[val])[:, 1]
        #if model_name == 'SVM':
          #    y_pred_prob = 1 - y_pred_prob
        fpr, tpr, thresholds = roc_curve(y_train.iloc[val], y_pred_prob)
        tprs.append(np.interp(mean_fpr, fpr, tpr))
        tprs[-1][0] = 0.0
        roc_auc = auc(fpr, tpr)
        aucs.append(roc_auc)
    # Plot ROC curve
    mean_tpr = np.mean(tprs, axis=0)
    mean_tpr[-1] = 1.0
    mean_auc = auc(mean_fpr, mean_tpr)
    std_auc = np.std(aucs)
    plt.plot(mean_fpr, mean_tpr, label=f'{model_name}', linewidth=2)
# Set plot properties
plt.plot([0, 1], [0, 1], linestyle='--', lw=2, color='gray', label='Reference', alpha=.8)
plt.xlim([0.0, 1.0])
plt.ylim([0.0, 1.0])
plt.gca().set_aspect('equal', adjustable='box')
plt.xlabel('1-Specificity', fontsize=16, labelpad=10)
plt.ylabel('Sensitivity', fontsize=16, labelpad=10)
plt.title('ROC Curves', fontsize=18, pad=15)
plt.legend(loc='lower right', fontsize=16)
plt.tight_layout()
plt.xticks(np.arange(0, 1.1, 0.1), fontsize=14)
plt.yticks(np.arange(0, 1.1, 0.1), fontsize=14)
# Display and save the plot
plt.savefig('roc_curves2.jpg', dpi=300)
plt.show()
metrics = {'Accuracy': accuracy_score, 'Sensitivity': recall_score}


for model_name, model in models.items():
    print(f"Evaluating {model_name}:")
    model.fit(X_train, y_train)
    y_pred = model.predict(X_test)
    for metric_name, metric in metrics.items():
        score = metric(y_test, y_pred)
        print(f"{metric_name}: {score:.3f}")
    tn, fp, fn, tp = confusion_matrix(y_test, y_pred).ravel()
    specificity = tn / (tn + fp)
    print(f"Specificity: {specificity:.3f}")
```

```
        # Compute ROC curve and AUC
        model_prob = CalibratedClassifierCV(model, cv=5)
        model_prob.fit(X_train, y_train)
        y_pred_prob = model_prob.predict_proba(X_test)[:, 1]
        fpr, tpr, thresholds = roc_curve(y_test, y_pred_prob)
        auc_score = auc(fpr, tpr)
        print(f"AUC: {auc_score:.3f}\n")


from sklearn.metrics import mean_squared_error
plt.figure(figsize=(6, 6))
brier_scores = {}
for model_name, model in models.items():
        print(f"Evaluating {model_name} (Calibration Curve):")
        # Calibration Curve calculations
        prob_true, prob_pred = [], []
        for train, val in cv.split(X_train, y_train):
                model.fit(X_train.iloc[train], y_train.iloc[train])
                y_pred_prob = model.predict_proba(X_train.iloc[val])[:, 1]
                fraction_of_positives, mean_predicted_value = calibration_curve(y_train.iloc[val], y_pred_prob, n_bins=10)
                prob_true.append(np.interp(np.linspace(0, 1, 10), mean_predicted_value, fraction_of_positives))
                prob_pred.append(np.linspace(0, 1, 10))
        # Plot Calibration Curve
        mean_prob_true = np.mean(prob_true, axis=0)
        mean_prob_pred = np.mean(prob_pred, axis=0)
        plt.plot(mean_prob_pred, mean_prob_true, label=model_name, marker='o')
        # Calculate Brier score
        brier_score = mean_squared_error(mean_prob_true, mean_prob_pred)
        brier_scores[model_name] = brier_score
        print(f"Brier Score for {model_name}: {brier_score:.4f}")
# Calibration curve formatting
plt.plot([0, 1], [0, 1], linestyle='--', lw=2, color='gray', label='Reference', alpha=.8)
plt.xlim([0.0, 1.0])
plt.ylim([0.0, 1.05])
plt.xlabel('Mean Predicted Probability', fontsize=14,labelpad=20)
plt.ylabel('Fraction of Positives', fontsize=14,labelpad=20)
plt.title('Calibration Curves', fontsize=16, y=1.05)
plt.legend(loc='lower right')
plt.xticks(np.arange(0, 1.1, 0.1), fontsize=14)
plt.yticks(np.arange(0, 1.1, 0.1), fontsize=14)
plt.legend(loc='lower right', fontsize=14, prop={'size': 14})
plt.show()


import numpy as np
import pandas as pd
from sklearn.linear_model import LogisticRegression
from sklearn.svm import SVC
```

```
from sklearn.ensemble import RandomForestClassifier
from sklearn.model_selection import train_test_split
from sklearn.metrics import roc_curve, roc_auc_score, accuracy_score, recall_score,
confusion_matrix
from sklearn.calibration import calibration_curve, CalibratedClassifierCV
import matplotlib.pyplot as plt
# Define the threshold probabilities for the DCA curve
thresh_probs = np.arange(0.1, 0.9, 0.1)
# Create an empty list to store the DCA scores for each model
dca_scores = []
# Define the cost-benefit matrix
# Here, we assume that a true positive (TP) result is worth 1, a false positive (FP) result
is worth -2,
# a false negative (FN) result is worth -1, and a true negative (TN) result is worth 0.
costbenefit = np.array([[0, -2], [-1, 1]])
# Define the feature and target variables
data = pd.read_excel(file_path)
X = data.drop(columns=['Group'])
y = data['Group']
# Split the data into training and test sets
X_train, X_test, y_train, y_test = train_test_split(X, y, test_size=0.2, random_state=42)
# Loop over the models and compute the DCA scores
for model_name, model in models.items():
    print(f"Evaluating {model_name}:")
    # Fit the model and make predictions on the test set
    model.fit(X_train, y_train)
    y_pred_prob = model.predict_proba(X_test)[:, 1]
    y_pred = (y_pred_prob >= 0.5).astype(int)
    # Compute the net benefit for a range of threshold probabilities
    net_benefit = []
    for thresh in thresh_probs:
        tn, fp, fn, tp = confusion_matrix(y_test, (y_pred_prob >=
thresh).astype(int)).ravel()
        net_benefit.append(np.sum(costbenefit * np.array([[fp, tp], [fn, tn]])) /
len(y_test))
    # Store the DCA scores for the model
    dca_scores.append(net_benefit)
    # Plot the DCA curve
    plt.plot(thresh_probs, net_benefit, label=model_name)
    plt.scatter(thresh_probs[np.argmax(net_benefit)], np.max(net_benefit), s=100,
marker='o', color='black')
    print(f"Optimal threshold for {model_name}:
{thresh_probs[np.argmax(net_benefit)]:.2f}")
    print(f"DCA score for {model_name}: {np.max(net_benefit):.3f}\n")
# Format the plot
plt.xlabel('Threshold Probability')
plt.ylabel('Net Benefit')
plt.title('Decision Curve Analysis')
plt.legend()
plt.show()
```

```
import numpy as np
import pandas as pd
from sklearn.linear_model import LogisticRegression
from sklearn.svm import SVC
from sklearn.ensemble import RandomForestClassifier
from sklearn.model_selection import train_test_split
from sklearn.metrics import confusion_matrix
import matplotlib.pyplot as plt
def calculate_net_benefit(thresh_group, y_pred_prob, y_label):
    net_benefit = []
    for thresh in thresh_group:
        y_pred_label = y_pred_prob > thresh
        tn, fp, fn, tp = confusion_matrix(y_label, y_pred_label).ravel()
        n = len(y_label)
        net_benefit.append(tp / n - fp / n * (thresh / (1 - thresh)))
    return net_benefit
def plot_dca(ax, thresh_group, net_benefit_model, color, model_name):
    # Plot the DCA curve
    ax.plot(thresh_group, net_benefit_model, color=color, lw=2, label=model_name)
    # Shade the area where the model has net benefit greater than treating all
    max_nb_idx = np.argmax(net_benefit_model)
    if net_benefit_model[max_nb_idx] > net_benefit_all[max_nb_idx]:
        ax.fill_between(thresh_group, net_benefit_model, net_benefit_all,
color=color, alpha=0.2)
    # Configure the plot aesthetics
    ax.set_xlim(0, 1)
    ax.set_ylim(-0.2, max(net_benefit_model) + 0.1)
    ax.set_xlabel('Threshold Probability', fontsize=22, fontweight='normal',
labelpad=20)
    ax.set_ylabel('Net Benefit', fontsize=22, fontweight='normal', labelpad=20)
    ax.set_title('Decision Curve Analysis', fontsize=24, fontweight='normal', y=1.05)
    ax.grid(True, alpha=0.5)
    ax.legend(loc='upper right', fontsize=24)
    return ax
# Define the feature and target variables
data = pd.read_excel(file_path)
X = data.drop(columns=['Group'])
y = data['Group']
# Split the data into training and test sets
X_train, X_test, y_train, y_test = train_test_split(X, y, test_size=0.2, random_state=42)
# Define the threshold probabilities for the DCA curve
thresh_group = np.arange(0, 1.01, 0.01)
# Calculate the net benefit for treating all patients
net_benefit_all = np.zeros_like(thresh_group)
tn, fp, fn, tp = confusion_matrix(y_test, np.ones_like(y_test)).ravel()
n = len(y_test)
net_benefit_all = tp / n - fp / n * (thresh_group / (1 - thresh_group))
# Loop over the models and compute the DCA curve
```

```
fig, ax = plt.subplots(figsize=(12, 10))
# Plot the Treat All and Treat None lines before the loop
ax.plot(thresh_group, net_benefit_all, color='gray', lw=2, linestyle='--', label='Treat All')
ax.plot([0, 1], [0, 0], color='gray', lw=2, linestyle=':', label='Treat None')
colors = [ 'blue','orange','green','red','purple',]
for i, (model_name, model) in enumerate(models.items()):
    print(f"Evaluating {model_name}:")
    # Fit the model and make predictions on the test set
    model.fit(X_train, y_train)
    y_pred_prob = model.predict_proba(X_test)[:, 1]
    # Calculate the net benefit for the model on the test set
    net_benefit_model = calculate_net_benefit(thresh_group, y_pred_prob, y_test)
    # Plot the DCA curve with a different color for each model
    ax = plot_dca(ax, thresh_group, net_benefit_model, color=colors[i], model_name=model_name)
    print(f"Optimal threshold for {model_name}: {thresh_group[np.argmax(net_benefit_model)]:.2f}")
    print(f"DCA score for {model_name}: {np.max(net_benefit_model):.3f}\n")
    # 设置横纵坐标刻度范围和间隔
plt.xticks(np.arange(0, 1.1, 0.1), fontsize=20)
plt.yticks(np.arange(0, 1.1, 0.1), fontsize=20)
plt.show()
   # Fit the model and make predictions on the test


fig.savefig("plotDCA1.jpg", dpi=300, bbox_inches='tight')


import pandas as pd
import numpy as np
from sklearn import svm
from sklearn.model_selection import train_test_split
from sklearn.preprocessing import StandardScaler
import shap
import matplotlib.pyplot as plt
import warnings
import tkinter as tk
from tkinter import filedialog
from PIL import Image
warnings.filterwarnings('ignore')
shap.initjs()


import matplotlib as mpl
mpl.rcParams['figure.dpi'] = 300


##models = {'Logit': log_reg, 'SVM': svm, 'RF': random_forest, 'XGBoost': xgb}
model2 = svm
```

```
explainer = shap.Explainer(model2, X_train, feature_names=data.columns[1:])
shap_values = explainer(X_test)


shap.plots.force(shap_values)


shap.plots.beeswarm(shap_values)
plt.savefig("beeswarm_plot.jpg", dpi=300, format='jpg')
plt.close()


shap.plots.bar(shap_values)
plt.savefig("bar_plot.jpg", dpi=300, format='jpg')
plt.close()


mean_shap_values = np.mean(np.abs(shap_values.values), axis=0)
max_contrib_index = np.argmax(mean_shap_values)
min_contrib_index = np.argmin(mean_shap_values)
max_contrib_feature = shap_values.feature_names[max_contrib_index]
min_contrib_feature = shap_values.feature_names[min_contrib_index]


shap.plots.force(shap_values[:, max_contrib_index])


shap.plots.force(shap_values[:, min_contrib_index])


root = tk.Tk()
root.withdraw()
file_path = filedialog.askopenfilename(filetypes=[("Excel files", "*.xlsx")])
data2 = pd.read_excel(file_path)


X_validation = data2.drop(columns=['Group'])
y_validation = data2['Group']


y_pred_prob = svm.predict_proba(X_validation)[:, 1]
#y_pred_prob = log_reg.predict_proba(X_validation)[:, 1]
#y_pred_prob = random_forest.predict_proba(X_validation)[:, 1]
fpr, tpr, thresholds = roc_curve(y_validation, y_pred_prob)
roc_auc = auc(fpr, tpr)
plt.figure(figsize=(6, 6))
plt.plot(fpr, tpr, label=f'SVM', linewidth=2)
plt.xlabel('False Positive Rate')
```

```
plt.ylabel('True Positive Rate')
plt.legend(loc='lower right')
# Set plot properties
plt.plot([0, 1], [0, 1], linestyle='--', lw=2, color='gray', label='Reference', alpha=.8)
plt.xlim([0.0, 1.0])
plt.ylim([0.0, 1.0])
plt.gca().set_aspect('equal', adjustable='box')
plt.xlabel('1-Specificity', fontsize=16, labelpad=10)
plt.ylabel('Sensitivity', fontsize=16, labelpad=10)
plt.title('ROC Curves', fontsize=18, pad=15)
plt.legend(loc='lower right', fontsize=16)
plt.tight_layout()
plt.xticks(np.arange(0, 1.1, 0.1), fontsize=14)
plt.yticks(np.arange(0, 1.1, 0.1), fontsize=14)
# Display and save the plot
plt.savefig('roc_curves_validtion.jpg', dpi=300)
plt.show()
metrics = {'Accuracy': accuracy_score, 'Sensitivity': recall_score}
roc_auc


prob_true, prob_pred = calibration_curve(y_validation, y_pred_prob, n_bins=10)
plt.figure(figsize=(6, 6))
plt.plot(prob_pred, prob_true, label='SVM', marker='o')
# Calibration curve formatting
plt.plot([0, 1], [0, 1], linestyle='--', lw=2, color='gray', label='Reference', alpha=.8)
plt.xlabel('Mean Predicted Probability', fontsize=14,labelpad=20)
plt.ylabel('Fraction of Positives', fontsize=14,labelpad=20)
plt.title('Calibration Curves', fontsize=16, y=1.05)
plt.legend(loc='lower right')
plt.xticks(np.arange(0, 1.1, 0.1), fontsize=14)
plt.yticks(np.arange(0, 1.1, 0.1), fontsize=14)
plt.legend(loc='lower right', fontsize=14, prop={'size': 14})
# Save the figure
plt.savefig('calibration_curve.jpg', dpi=300, bbox_inches='tight')
plt.show()


def calculate_net_benefit(thresh_group, y_pred_prob, y_label):
    net_benefit = []
    for thresh in thresh_group:
        y_pred_label = y_pred_prob > thresh
        tn, fp, fn, tp = confusion_matrix(y_label, y_pred_label).ravel()
        n = len(y_label)
        net_benefit.append(tp / n - fp / n * (thresh / (1 - thresh)))
    return net_benefit
def plot_dca(ax, thresh_group, net_benefit_model, color, model_name):
    # Plot the DCA curve
    ax.plot(thresh_group, net_benefit_model, color=color, lw=2, label=model_name)
    # Shade the area where the model has net benefit greater than treating all
```

```
    max_nb_idx = np.argmax(net_benefit_model)
    if net_benefit_model[max_nb_idx] > net_benefit_all[max_nb_idx]:
        ax.fill_between(thresh_group, net_benefit_model, net_benefit_all,
color=color, alpha=0.2)
    # Configure the plot aesthetics
    ax.set_xlim(0, 1)
    ax.set_ylim(-0.2, max(net_benefit_model) + 0.1)
    ax.set_xlabel('Threshold Probability', fontsize=22, fontweight='normal',
labelpad=20)
    ax.set_ylabel('Net Benefit', fontsize=22, fontweight='normal', labelpad=20)
    ax.set_title('Decision Curve Analysis', fontsize=24, fontweight='normal', y=1.05)
    ax.grid(True, alpha=0.5)
    ax.legend(loc='upper right', fontsize=24)
    return ax
# Define the threshold probabilities for the DCA curve
thresh_group = np.arange(0, 1.01, 0.01)
# Calculate the net benefit for treating all patients
net_benefit_all = np.zeros_like(thresh_group)
tn, fp, fn, tp = confusion_matrix(y_validation, np.ones_like(y_validation)).ravel()
n = len(y_validation)
net_benefit_all = tp / n - fp / n * (thresh_group / (1 - thresh_group))
# Compute DCA curve
fig, ax = plt.subplots(figsize=(12, 10))
# Plot the Treat All and Treat None lines before the loop
ax.plot(thresh_group, net_benefit_all, color='gray', lw=2, linestyle='--', label='Treat
All')
ax.plot([0, 1], [0, 0], color='gray', lw=2, linestyle=':', label='Treat None')
# Calculate the net benefit for the model on the validation set
net_benefit_model = calculate_net_benefit(thresh_group, y_pred_prob, y_validation)
# Plot the DCA curve with a different color for each model
ax = plot_dca(ax, thresh_group, net_benefit_model, color='red', model_name='SVM')
print(f"Optimal threshold for XGB:
{thresh_group[np.argmax(net_benefit_model)]:.2f}")
print(f"DCA score for XGB: {np.max(net_benefit_model):.3f}\n")
plt.xticks(np.arange(0, 1.1, 0.1), fontsize=20)
plt.yticks(np.arange(0, 1.1, 0.1), fontsize=20)
plt.show()
fig.savefig("plotDCA_validation.jpg", dpi=300, bbox_inches='tight')
```
